# A novel 3D image registration technique for augmented reality vision in minimally invasive thoracoscopic pulmonary segmentectomy

**DOI:** 10.1007/s11548-024-03308-7

**Published:** 2024-12-20

**Authors:** J. J. Peek, X. Zhang, K. Hildebrandt, S. A. Max, A. H. Sadeghi, A. J. J. C. Bogers, E. A. F. Mahtab

**Affiliations:** 1 Department of Cardiothoracic Surgery, Erasmus University Medical Center, Rotterdam, The Netherlands; 2 Computer Vision Lab, TU Delft, Delft, The Netherlands; 3 Computer Graphics and Visualization Lab, TU Delft, Delft, The Netherlands; 4 Department of Cardiothoracic Surgery, University Medical Center Utrecht, Utrecht, The Netherlands; 5 Department of Cardiothoracic Surgery, Leiden University Medical Center, Leiden, The Netherlands

**Keywords:** Augmented reality, 3D point cloud, Registration, Video-assisted thoracoscopic surgery, Robotic-assisted thoracoscopic surgery

## Abstract

**Purpose:**

In this feasibility study, we aimed to create a dedicated pulmonary augmented reality (AR) workflow to enable a semi-automated intraoperative overlay of the pulmonary anatomy during video-assisted thoracoscopic surgery (VATS) or robot-assisted thoracoscopic surgery (RATS).

**Methods:**

Initially, the stereoscopic cameras were calibrated to obtain the intrinsic camera parameters. Intraoperatively, stereoscopic images were recorded and a 3D point cloud was generated from these images. By manually selecting the bifurcation key points, the 3D segmentation (from the diagnostic CT scan) was registered onto the intraoperative 3D point cloud.

**Results:**

Image reprojection errors were 0.34 and 0.22 pixels for the VATS and RATS cameras, respectively. We created disparity maps and point clouds for all eight patients. Time for creation of the 3D AR overlay was 5 min. Validation of the point clouds was performed, resulting in a median absolute error of 0.20 mm [IQR 0.10–0.54]. We were able to visualize the AR overlay and identify the arterial bifurcations adequately for five patients. In addition to creating AR overlays of the visible or invisible structures intraoperatively, we successfully visualized branch labels and altered the transparency of the overlays.

**Conclusion:**

An algorithm was developed transforming the operative field into a 3D point cloud surface. This allowed for an accurate registration and visualization of preoperative 3D models. Using this system, surgeons can navigate through the patient's anatomy intraoperatively, especially during crucial moments, by visualizing otherwise invisible structures. This proposed registration method lays the groundwork for automated intraoperative AR navigation during minimally invasive pulmonary resections.

**Supplementary Information:**

The online version contains supplementary material available at 10.1007/s11548-024-03308-7.

## Introduction

Lung cancer is one of the leading causes of cancer-related mortality worldwide [1]. For clinical stage 1A non-small cell lung cancer (NSCLC) with tumor size of two cm or less, segmentectomy procedure shows positive short- and long-term outcomes [2]. However, segmentectomy is a technically more challenging procedure than a lobectomy, especially when combined with a video or robotic-assisted thoracoscopic surgery (VATS/RATS) approach. To address these challenges, the current expert consensus of the European Society of Thoracic Surgeons (ESTS) recommends preoperative 3D reconstructions for preparing segmentectomy surgery, particularly for tumor localization, recognizing anatomical variations, and estimating resection margins [3]. However, the intraoperative position and orientation of the lung are completely different compared to the preoperative insufflated lung, since the lungs are deflated and manipulated by the surgeon to facilitate the operation. This altered pulmonary state, together with extensive anatomical variations in bronchi and vasculature, makes it challenging to predict the intraoperative location and relationship between the broncho-vasculature and tumor.

Intraoperative augmented reality (AR) overlays can aid in identifying structures and visualizing hidden structures, for example by augmenting the surgical view with the preoperative 3D model derived from a computed tomography (CT) scan [4–6]. However, a major technical hurdle in facilitating intraoperative AR visualization is the registration (i.e., aligning) of deformable organs with the corresponding 3D models. Image registration is the technique of aligning multiple images or 3D volumes to the same coordinate system [7]. Registration is challenging due to the high deformability, limited visible contours and features, and often only partially visibility of the organ during surgery [4, 5]. We could potentially leverage surface-based registration using an intraoperative point cloud, or anatomical landmark-based registration with landmarks such as vascular bifurcations, organ ridges, or shapes for 3D registration [4, 5]. Moreover, the creation of an intraoperative point cloud or (automatic) selection of landmarks is complex due to repetitive textures and the lack of distinct image features [5]. Intraoperatively, only a small part of the broncho-vascular anatomy is visible, making partial to whole registration necessary. This process is difficult to automate, as the small part of the anatomy could fit the structure of the lung in many different ways. For performing a pulmonary segmentectomy, the broncho-vasculature is more critical than the lung parenchyma itself compared to other deformable organs where AR overlays can be applied [4–6], as it includes dissection and stapling of the segmental bifurcations [3]. Registration could also be performed manually and merged using video-mixing software [6]; however, it is labor-intensive and an anatomical/technical expert is required to perform the live registration updates of the organ position.

In this work, we aim to assess the feasibility of creating a dedicated pulmonary VATS/RATS AR workflow to enable a semi-automated intraoperative overlay of the pulmonary anatomy. Specifically, we have matched the preoperative 3D segmentation derived from the diagnostic CT scan to the 3D point cloud derived from stereoscopic VATS and RATS images. Registration is performed using a combination of surface and anatomical landmark registration, using vascular bifurcations. With the proposed AR technique, we expect to optimally guide the surgeons applying the 3D models intraoperatively. To our knowledge, this study represents the first application of these techniques in the context of AR-based pulmonary surgery.

## Methods

**Fig. 1:**
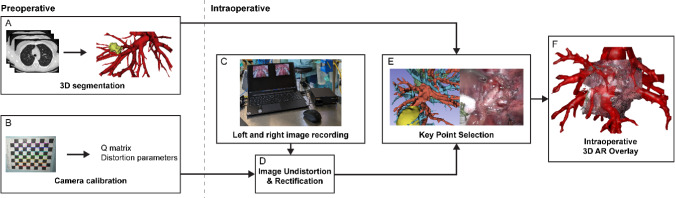
Overview of the proposed workflow for generating a preoperative 3D reconstruction, intraoperative 3D point cloud, and 3D AR overlay. Initially, preoperative 3D segmentation is performed (**A**), and camera calibration parameters are obtained through camera calibration for distortion and rectification of the left and right images (**B**, **D**). Stereoscopic images are recorded (**C**) and a 3D point cloud is created of the visible intraoperative surface, using the camera calibration parameters. Corresponding key points were selected on both the preoperative 3D model and the intraoperative 3D point cloud (**E**). Subsequently, the preoperative 3D model was registered onto the 3D point cloud. Finally, an intraoperative AR overlay was created during VATS/RATS pulmonary resection procedures (F). AR = Augmented Reality, VATS = Video Assisted Thoracic Surgery, RATS = Robotic Assisted Thoracic Surgery

This research was approved by the medical ethical committee of the Erasmus University Medical Center (MEC-2023–0080). We created a stepwise workflow for applying surface and anatomical landmark-based registration for creating AR overlays as shown in Fig. 1.

### Patient population

We selected 4 patients undergoing segmentectomy and 4 patients undergoing lobectomy via VATS or RATS. Tumors were located in different lobes; 3 right lower lobe resections, 2 right upper lobe resections, 2 left lower lobe resections, and 1 left upper lobe resection were included and recorded for investigating the feasibility of our proposed workflow. All included patients provided written informed consent.

### Camera calibration

First, stereo camera calibration was performed of both the VATS and RATS stereoscopic cameras (Fig. 1B) [8]. We used a checkerboard with 9 × 7, 6 mm squared pattern to obtain the intrinsic camera parameters of the stereoscopic image pairs, presented as a reprojection matrix Q [9], including the focal length f [pixels], principal point of the left camera (c_x1_, c_y_)[pixels], principal point of the right camera c_x2_ [pixels], and the baseline length T_x_:

1 $$ Q = \left( {\begin{array}{*{20}c} 1 & \quad  0 &  \quad 0 &  \quad { - c_{x1} } \\ 0 &  \quad 1 & \quad  0 & \quad  { - c_{y} } \\ 0 &  \quad 0 &  \quad 0 & \quad  f \\ 0 &  \quad 0 & \quad  { - \frac{1}{{T_{x} }}} &  \quad {\frac{{c_{x1} - c_{x2} }}{{T_{x} }}} \\ \end{array} } \right) $$

### Image acquisition

**Fig. 2:**
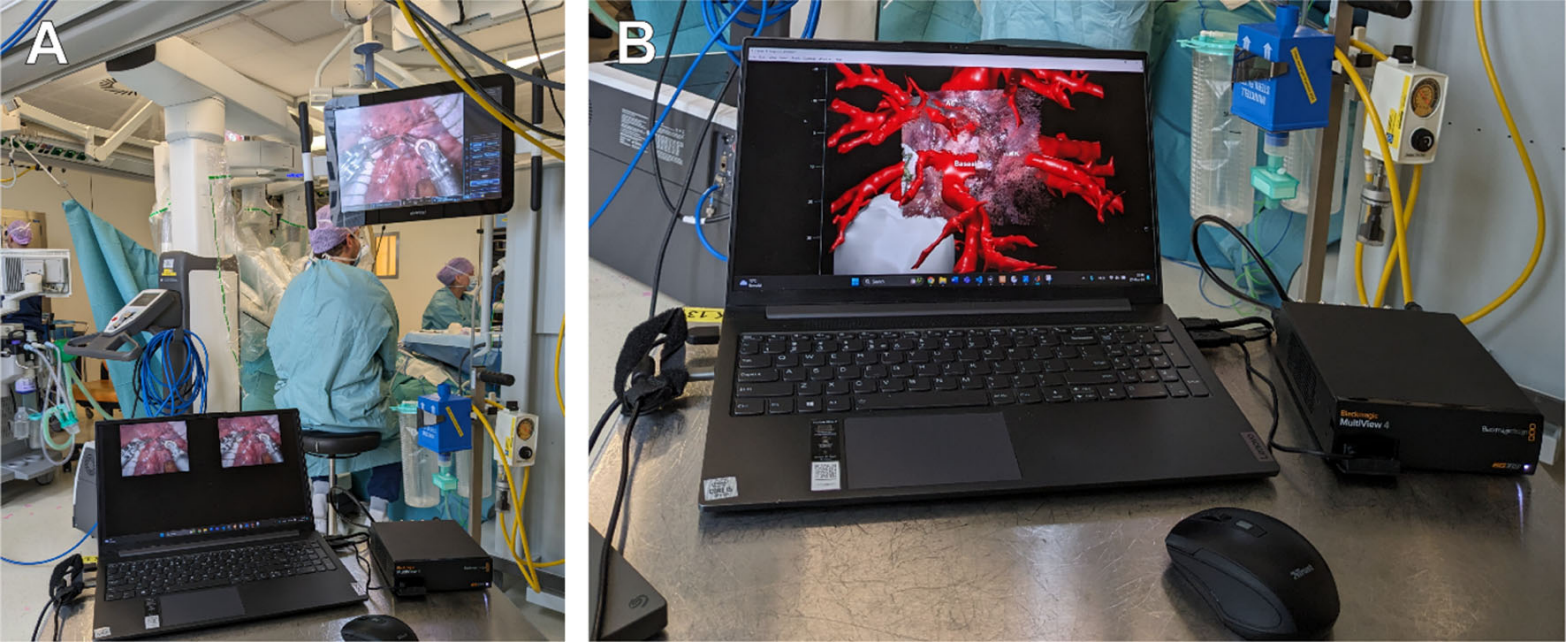
Recording setup. **A** We used the Blackmagic MultiView 4 and Elgato Cam Link 4 k to record both the left and the right eye of the stereoscopic camera simultaneously. **B** Intraoperative augmented reality overlay using our proposed AR lung application. The overlay can be created onto the 3D point cloud of the operative scene with a slight delay, allowing to be directly visualized via the TilePro application during RATS procedures. *RATS* Robotic-assisted thoracic surgery, *AR* augmented reality, *VATS* video-assisted thoracic surgery

Preoperatively for surgical planning, 3D mesh models were created of the broncho-vasculature and tumor, using artificial intelligence (AI) and semiautomatic segmentation (Fig. 1A) [10]. Intraoperatively, images were acquired of a view on the arterial segmental branches of the RATS (DaVinci 30° 3DHD endoscope, Intuitive, Sunnyvale, CA, USA) and VATS (TIPCAM1 Rubina 3D 4 K 30° endoscope, Karl Storz, Tuttlingen, Germany) thoracoscopic cameras. Image acquisition was performed using the Blackmagic Multiview 4 (Blackmagic Design, Port Melbourne, Australia) and Elgato Cam Link 4 K (Corsair, Milpitas, CA, USA) (Fig. 1C**, **Fig. 2). The VATS images needed an additional preprocessing step for de-interlacing the even and odd rows and separate the left and right eye images, using Python 3.9.12 [11]. All left and right eye images were undistorted and rectified with the intrinsic calibration parameters via the OpenCV package in Python 3.9.12 (Fig. 1D) [12]. The undistorted images were saved and imported in MATLAB R2023b (The MathWorks Inc, Natick, MA, USA) for point cloud creation and registration.

### 3D point cloud creation

A disparity map was created from the corresponding undistorted left and right images [13]. Subsequently, the 3D point cloud was created by calculating the real-world 3D points and depth of each pixel from the disparity map, using the intrinsic camera calibration parameters [14]. These 3D points are combined with the left image for the color corresponding to the 3D points and can be visualized in a 3D plot.

### Initial registration

**Fig. 3:**
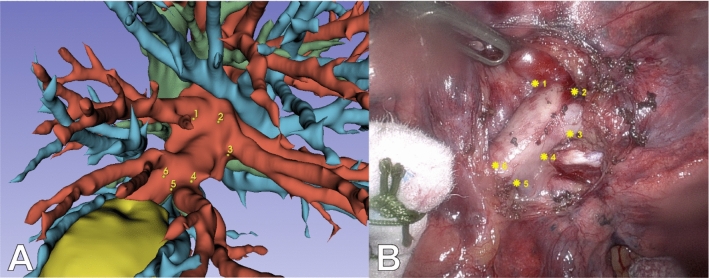
Applied key points using bifurcation landmarks on the preoperative model (**A**) and intraoperative image (**B**)

The preoperative 3D meshes of the arteries, veins, bronchi, and tumor used for surgical planning were saved as separate meshes (**P**(F,V)) with faces (F) and vertices (V). 3D key points of visible bifurcations (k_preop_) were first manually selected on the preoperative 3D model using Slicer 3D 5.6.0 (Fig. 1E, Fig. 3A) [15]. The same number of corresponding key points was selected on the intraoperative image, at the bifurcations of the visible arterial branches (k_intraop_) (Fig. 3B). These point pairs were used to calculate the optimal rotation and translation matrices of the preoperative 3D model (moving) onto the intraoperative 3D point cloud (fixed) [16, 17]. Finally, the intraoperative 3D point cloud and transformed 3D mesh model were visualized together as an AR overlay (Fig. 1F). For the RATS procedures, the AR overlay could be directly visualized in the robotic console using the TilePro application.

### Feasibility

The 3D point cloud results were validated by measuring the diameter of vascular structures on the 3D point cloud and comparing them with the diameter of the corresponding vessel on the CT scan. Moreover, the size of visible instruments was measured on the 3D point cloud and compared with the physical dimensions of the instrument. The median absolute error of the point cloud measurements was calculated to assess feasibility and accuracy of the 3D point clouds. The landmark registration error was calculated as a root mean squared distance (RMSD) between the intraoperative and preoperative landmark sets. Furthermore, AR overlays were considered successful if the intraoperatively visible bifurcations of the preoperative 3D model were correctly aligned to the corresponding branches visible during surgery. An overlay was deemed unsuccessful if these bifurcations were misaligned, for example the direction of the intraoperative visible artery was different from the direction of the projected branch, or if the preoperative 3D model was not matching with the intraoperative anatomy.

## Results

### Camera calibration

We obtained the following two Q matrices for the VATS and RATS stereoscopic camera pairs, respectively:

$$\begin{aligned} Q_{{{\text{VATS}}}} &= \left( {\begin{array}{*{20}c} 1 & \quad  0 & \quad  0 & \quad  { - 988.53} \\ 0 &  \quad 1 & \quad 0 \quad  & { - 546.98} \\ 0 &  \quad 0 &  \quad 0 &  \quad {1432.8} \\ 0 &  \quad 0 &  \quad {0.16406} &  \quad 0 \\ \end{array} } \right),\\  Q_{{{\text{RATS}}}} &= \left( {\begin{array}{*{20}c} 1 &  \quad 0 &  \quad 0 &  \quad { - 672} \\ 0 &  \quad 1 &  \quad 0 &  \quad { - 523.24} \\ 0 &  \quad 0 &  \quad 0 &  \quad {1013.3} \\ 0 &  \quad 0 &  \quad {0.25266} &  \quad 0 \\ \end{array} } \right) \end{aligned}$$

The 3D points of the calibration pattern were reprojected back to the original 2D images, which resulted in the overall mean reprojection error of 0.34 pixels for the VATS camera (1080 × 1920 pixel resolution) and 0.22 pixels for the RATS camera (1010 × 1264 pixel resolution).

### Point cloud

**Fig. 4:**
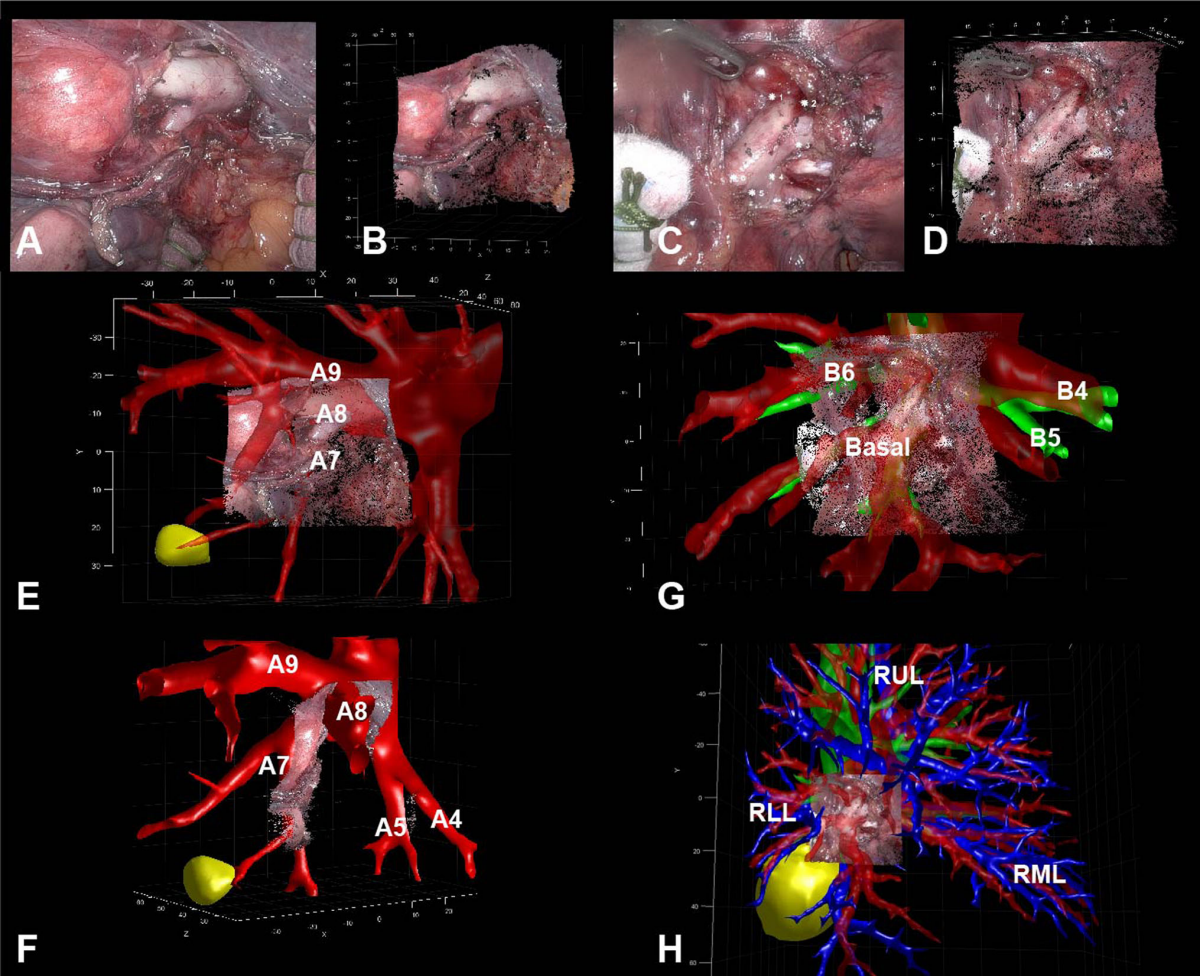
Intraoperative view, 3D point clouds and 3D overlays **A** Intraoperative left-eye image of a patient undergoing a segment 7 resection via RATS, **B** front view of the corresponding point cloud. **C** Intraoperative left-eye image of a patient undergoing a right lower lobe resection via RATS, **D** front view of the corresponding point cloud. **E** The arterial bifurcations are correctly registered and labeled, the artery to segment 7 (A7), segment 8 (A8) and segment 9 (A9) are clearly visible. The tumor is shown in yellow, and is located in segment 7. **F** The 3D point cloud with overlay is rotated clockwise, the middle lobe arteries (A4 and A5) are visible, the two main branches of A7 are clearly visible below the intraoperative visible surface. A8 is directed towards the user in this angle. A9 is directed inside the intraoperative view and therefore cannot be visualized fully on the intraoperative image. **G** The arteries are shown transparently, and the bronchus is shown behind the artery and intraoperative surface. The bronchus branches to segment 6 (B6), the right middle lobe (B4 and B5) and basal bronchi (basal) are well visible through the transparent artery surface. **H** Zoomed out overview of the full preoperative anatomy (artery, vein, bronchus, tumor) with respect to the intraoperative visible surface. Arteries are indicated in red, veins are indicated in blue, bronchus is indicated with green, tumor is indicated in yellow. Please refer to the Supplementary video for a video of these intraoperative 3D point clouds and AR overlays. RATS = Robot Assisted Thoracic Surgery, RUL = right upper lobe, RML = right middle lobe, RLL = right lower lobe

With images from calibrated cameras, 3D point cloud surfaces were created, showing an adequate 3D model of the surgical field (Fig. 4A, B, C, D). We were able to create a disparity map and subsequent 3D point cloud surface of the recordings of all eight patients’ artery bifurcations. Arterial and venous diameters were measured and compared to the diameters on the CT scan, and the thoracoscopic instruments were measured and compared with their physical size. The median absolute error between the actual and measured values was 0.20 mm [IQR 0.10–0.54]. Next, initial rigid registration and overlay visualization were successful in five out of eight patients, using 6 [range: 5–10] anatomical landmarks on average, with a visibility of 4 [range: 3–6] arterial bifurcations (Table 1). The landmark registration errors were calculated, resulting in an average RMSD of 6.6 mm [range: 4.45–9.6].

**Table 1:** Included patient cases

Patient ID	Surgery	Lobe	Surgical approach	# Visible branches [n]	# Fiducials [n]	RMSE [mm]	Successful 3D AR overlay	Comment
1	Segmentectomy S8	RLL	VATS	6	6	5.833	Yes	–
2	Lobectomy	LLL	RATS	6	10	9.602	Yes	–
3	Bi-segmentectomy S1 and S3	RUL	RATS	4	5	6.173	No	Distorted arterial anatomy + overlapping instruments
4	Segmentectomy S7	RLL	RATS	4	6	6.458	Nes	–
5	Segmentectomy S1 and S2	RUL	RATS	2	-	-	No	3D model and intraoperative anatomy unequal
6	Lobectomy	LUL	RATS	3	4	4.459	No	Distorted arterial anatomy + dirty camera lens
7	Lobectomy	RLL	RATS	4	6	6.041	Yes	–
8	Lobectomy	LLL	RATS	3	5	8.071	Yes	–

**Fig. 5:**
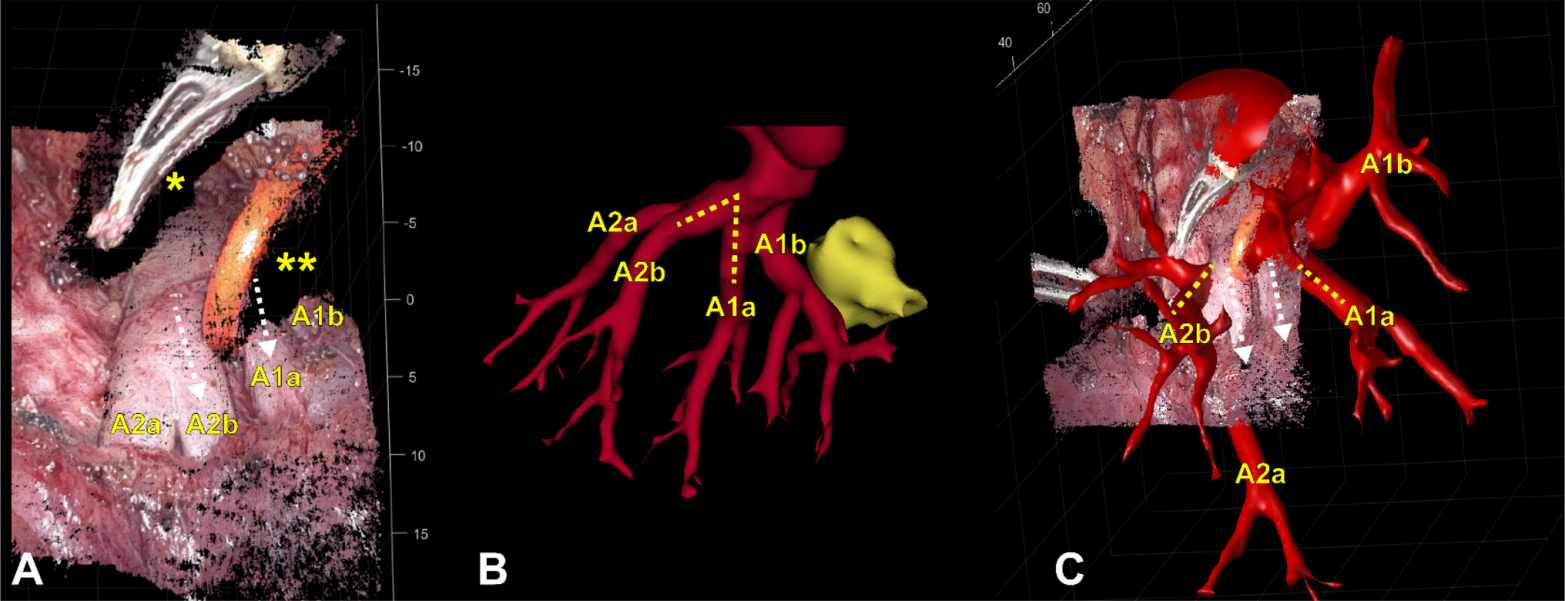
Intraoperative 3D point cloud without accurate 3D overlay**. A** Intraoperative point cloud of a patient undergoing bi-segmentectomy of segments 1 and 3 of the RUL. Arteries to segment 1(A1a, A1b) and segment 2 (A2a, A2b) are intraoperatively visible. However, parts of the artery are not visible in the 3D point cloud, because of the instrument (marked with *) and the vessel loop (marked with **) which are in line of sight between the camera and the arteries. **B** 3D model of the intraoperatively visible arteries (red). The tumor (yellow) and located in segment 1. It is clearly visible that the angle between the A1a and A2b arteries is much smaller intraoperatively, as the arteries are more or less parallel, because manipulation of the RUL by the surgeon. **C** Inaccurate 3D AR overlay, as the A2a, A2b, A1a, and A1b are all parallel on the intraoperative view; however, on the 3D overlay, A2a is directed into the surgical field, A2b is directed toward the stereoscopic camera and A1a is directing in an angle of the actual A1a. *RUL* right upper lobe

The visible arteries were correctly identified and overlaid onto the intraoperatively recorded image with the full model of the artery, using our aforementioned registration method. Overlapping instruments together with insufficient image quality (dirty camera lens, bloody image with limited features) caused an unsuccessful registration for two patients. When the image quality of the input image was insufficient, the point cloud did have a least limited defined surface and more outliers, because of fewer distinguishable features in the image, resulting in increased noise and outliers and complicating adequate landmark selection and registration. Furthermore, when objects such as instrument, vessel loop, or gauze obstructed the line of sight between the camera and the artery, this made point cloud creation of the concealed structure impossible, and the resulting point cloud will include gaps. Figure 5 shows an example of a failed 3D AR overlay, with gaps in the point cloud close to the artery (Fig. 5A). Moreover, the intraoperative anatomy is different as the angle between the different arterial branches is decreased compared to preoperatively (Fig. 5B, C). Additionally, the AI-based segmentation had mislabeled an artery as a vein, making registration unfeasible in one patient. Besides the intraoperatively visible arterial branches, we were able to visualize and superimpose hidden structures (vein, tumor, bronchus) onto the intraoperative images for these five patients. These structures were visible underneath the surface of the intraoperative 3D point cloud (Fig. 4E, F, G, H). Besides creating AR overlays, we were able to visualize branch labels and could adjust the transparency for visualization of multiple overlapping structures, for example, a bronchial branch underneath the arterial branch (Fig. 4F). To avoid visual distractions, the view could be cropped such that only the tumor and nearby branches of the 3D model were shown, and it was possible to toggle the visibility of individual 3D structures (tumor, bronchus, artery, vein). Additionally, it was possible to zoom out and show the full preoperative 3D model with respect to the intraoperative 3D point cloud, providing the surgeon with some contextual awareness (Fig. 4H). Using this visualization technique, surgeons could see what is situated below the intraoperative surface (Supplementary Video). The creation of these overlays currently took approximately 5 min, making it feasible to run the algorithm with a slight delay during the procedure.

## Discussion

In this manuscript, we propose a method for developing an intraoperative 3D point cloud from stereoscopic VATS and RATS images. This method allows creating 3D models of the intraoperative surface, with a median error of 0.20 mm. Using these 3D point clouds, we were able to apply a registration algorithm and create an accurate intraoperative 3D AR overlay of the visible arterial bifurcations onto the surgical field in 5/8 patient cases. Besides an overlay of visible bifurcations, we could localize and visualize intraoperatively invisible structures, such as the tumor hidden in the parenchyma, or venous or bronchial branches hidden behind a visible artery. Using these intraoperative models, the surgeons could be guided through the surgery. This could potentially aid the surgeons promptly identifying the arteries (to ligate or to preserve) and locating the tumor.

Some limitations and software improvements were identified. Our method applies rigid registration for creating accurate overlays of the lung hilum onto the intraoperative field. However, the intraoperative deformation in the lungs is non-rigid including both manipulation of the surgeons and deflation of the lung. This anatomy difference between the pre- and intraoperative situation caused a failed AR overlay in two cases (Fig. 5). We achieved a correct overlay for the visible branches in five patient cases. Nonetheless, accuracy for structures or branches located further from the lung hilum (i.e., more peripheral and distant from the intraoperative visible surgical field) remains unknown and is likely less accurate due to intraoperative deformations and collapse. Rigid registration as proposed in this paper can be applied for initial registration, after which non-rigid surface registration is suggested including the intraoperative deformations, and probably improving the registration accuracy [18]. However, the high flexibility and collapse of the lung make orientation and registration more complex than in other deformable organs, such as the kidney or liver, where the parenchyma could be used for surface registration. Further research should focus on investigating the deformation of the broncho-vasculature during this collapse and improve dynamical pulmonary models potentially used as 3D input for the registration [19]. Currently, visible bifurcations are needed for intraoperative orientation and registration. Ideally, surgeons would like to know where to target the dissection before opening and dissecting the tissue, to enhance safety and efficacy. Applying a dynamic model of a collapsed lung could improve the correspondence between the pre- and intraoperative anatomy, and could potentially enable parenchyma-based registration, visualizing hidden structures before dissection of the vasculature. In this study, we confirmed that occlusion of instruments close to the registration target can impede registration because of missing point cloud data (Fig. 5). Recording clean images, showing as much as the anatomical target as possible, without instruments in the line of sight is essential [20]. Moreover, improving the disparity map and 3D point clouds could also aid improving the registration, by image preprocessing, noise reduction, and removal of outliers [21]. Additionally, instruments can be filtered out using AI algorithms [22], or algorithms accounting for missing information in the 3D point cloud could be employed, such as super-pixel segmentation, combining pixels with comparable or continuous depths or applying AI algorithms for endoscopic point cloud completion [23, 24].

Furthermore, in this study we presented the landmark registration accuracy as RSMD, but this accuracy value is not correlating with an adequate visible overlay, and we showed that the landmark registration error can be significant, while still having a an adequate intraoperative overlay, and vice versa. In addition to deformations, the error includes manually point selection inaccuracies, depending on point visibility in both images; therefore, the RSMD is an unspecific parameter for the quality of the registration [25]. An alternative parameter should be used to assess the accuracy and validate the registration. For example, the amount of overlap between point clouds could be used [26]. However, an optimal validation method is yet to be discovered, as often no ground truth exists in these type of registration problems [25, 27]. Moreover, an essential question for clinical implementation is determining the threshold for an acceptable overlay accuracy, which is a topic not adequately addressed yet in the current literature [18].

Ideally, the visualization of the AR overlays should be able to be performed intraoperatively with no or minimal interruption of the surgical workflow. Currently, the workflow is not yet real time, as creation of these overlays takes approximately 5 min *after* recording of the intraoperative video. To reduce the processing time, more automation of the registration method is needed, using automated detection and registration of structures and bifurcation landmarks using advanced AI algorithms [18]. Concurrently to automated registration, dynamic tracking should be integrated and automated in the software for real-time marker-less tracking. Techniques such as simultaneous localization and mapping (SLAM) combined with iterative closest point (ICP) algorithms, previously applied for kinect fusion [28] or thin-plate splines (TPS) models, which have been applied for tracking the heart motion in minimally invasive cardiac surgery [29], could be adapted for the purpose of dynamic tracking.

For clinical implementation, a graphical user interface (GUI) is crucial, enabling easy interaction and toggling visibility of structures. Surgeons could use it independently, or during the implementation phase, a technician who is also familiar with the intraoperative anatomy could assist in performing the registration and displaying the AR overlay. Ultimately, this GUI will be fully incorporated into the robotic surgical console or be applied while scrubbed in (using a sterile tablet or hand tracking devices) [30]. Finally, it is essential to assess the clinical applicability of the proposed AR overlay method, by a validation study with increased sample, quantifying system safety and overlay accuracy [27]. Moreover, the intraoperative utility should be assessed by employing validated useability questionnaires, as well as the effect on clinical outcomes and learning curves.

## Conclusion

We proposed an algorithm transforming the operative field into a 3D point cloud surface. On top of this 3D point cloud surface, our method can adequately register and visualize preoperative 3D models. Using this system, surgeons can be navigated through the patient's anatomy intraoperatively during crucial moments, by visualizing otherwise invisible structures hidden underneath the surface. This method is forming the foundation for automatically registered intraoperative AR navigation during VATS/RATS pulmonary resections, highlighting the potential and feasibility for developing intraoperative 3D-AR guidance for patients undergoing VATS/RATS segmentectomy. Some improvements for visualization and registration are suggested in this paper. Clinical and technical validation of our proposed registration method for AR pulmonary surgery is mandatory prior to clinical implementation.

## Supplementary Information

Below is the link to the electronic supplementary material.

Supplementary file1 (MP4 193851 KB)
